# Neurosonological Examination: A Non-Invasive Approach for the Detection of Cerebrovascular Impairment in AD

**DOI:** 10.3389/fnbeh.2014.00004

**Published:** 2014-01-21

**Authors:** Barbora Urbanova, Ales Tomek, Robert Mikulik, Hana Magerova, Daniel Horinek, Jakub Hort

**Affiliations:** ^1^Department of Neurology, 2nd Faculty of Medicine, Motol University Hospital, Charles University, Prague, Czech Republic; ^2^Department of Neurology, International Clinical Research Center, St. Anne’s University Hospital, Brno, Czech Republic; ^3^Department of Neurosurgery, 1st Faculty of Medicine, Central Military Hospital, Charles University, Prague, Czech Republic; ^4^International Clinical Research Center, St. Anne’s University Hospital, Brno, Czech Republic

**Keywords:** Alzheimer’s disease, carotid ultrasound, cerebrovascular reserve capacity, neurosonology, transcranial ultrasound

## Abstract

There has been a growing interest in vascular impairment associated with Alzheimer’s disease (AD). This interest was stimulated by the findings of higher incidence of vascular risk factors in AD. Signs of vascular impairment were investigated notably in the field of imaging methods. Our aim was to explore ultrasonographic studies of extra- and intracranial vessels in patients with AD and mild cognitive impairment (MCI) and define implications for diagnosis, treatment, and prevention of the disease. The most frequently studied parameters with extracranial ultrasound are intima-media thickness in common carotid artery, carotid atherosclerosis, and total cerebral blood flow. The transcranial ultrasound concentrates mostly on flow velocities, pulsatility indices, cerebrovascular reserve capacity, and cerebral microembolization. Studies suggest that there is morphological and functional impairment of cerebral circulation in AD compared to healthy subjects. Ultrasound as a non-invasive method could be potentially useful in identifying individuals in a higher risk of progression of cognitive decline.

## Vascular Changes and Alzheimer’s Disease

In an effort to reveal an etiopathogenic mechanism responsible for Alzheimer’s disease (AD) many hypotheses have been postulated. Recent failures of candidate disease-modifying medications have led to many alternative theories of AD pathophysiology. Multiple studies suggest that the risk of AD is apart from other factors associated with midlife hypertension, diabetes mellitus, hypercholesterolemia, and other vascular risk factors (Breteler, 2000; Casserly and Topol, 2004; Gorelick, 2004; Shah et al., 2012). This association has led to a hypothesis that the vascular risk factors could play an important role in the genesis or in the progression of the disease, but even after years of research the role of vascular risk factors in AD remains a subject of discussion. Two principal theories were postulated. First, an impaired cerebral circulation from any cause leads to neurodegeneration (de la Torre, 2010). Second, vascular impairment from any cause (e.g., atherosclerosis) accelerates the rate of progression of neurodegeneration (Kalaria, 2002). The second theory is generally more accepted.

Various imaging methods were used to explore the signs of vascular impairment in AD. White matter lesions in people above 65 years are associated with typical vascular risk factors and cognitive decline (Breteler et al., 1994; DeCarli et al., 2001; Wu et al., 2002). Higher extent of white matter lesions in MCI patients is associated with higher risk of progression of MCI to dementia of any kind (Wolf et al., 2000). In MCI patients, there is a regional hypoperfusion on SPECT examination in hippocampus, amygdala, and prefrontal cortex (Johnson et al., 1998), and in AD patients the perfusion is decreased in whole temporoparietal region and correlates with the disease severity (DeKosky et al., 1990) [AD patient with varying disease severity were divided into four groups according to mini-mental state examination (MMSE): >24; 22–24; 15–21; <15].

The objective of this review was to explore extracranial and transcranial ultrasound projects in AD patients. We tried to describe the pattern of functional or structural cerebrovascular impairment in AD as characterized by ultrasonography, and to summarize ultrasound parameters of cerebral circulation in AD vs. healthy control subjects or in AD patients longitudinally. We have discussed to what extent neurosonological examination could contribute to diagnosis, prevention, or treatment of AD. We have also discussed whether there is a special pattern of circulation impairment, namely: Is AD associated with large vessel or, rather, small vessel disease? Are there predominant changes in a specific region of the brain? Is the incidence of microembolization higher in AD, or is there a correlation of any parameter with disease progression?

## Extracranial Ultrasound in AD

Main parameters that can be assessed by extracranial ultrasound are parameters of arterial wall [carotid intima-media thickness (IMT) and atherosclerotic plaques] and cerebral perfusion [total cerebral blood flow (CBF)].

### IMT and carotid atherosclerosis

Carotid IMT is defined as a distance between media–adventitia interface and intima–lumen interface measured on the common carotid artery, 1–2 cm proximally from bifurcation or, less frequently, on the internal carotid artery using automated analyzers implemented in most of the recent ultrasound devices. IMT is generally regarded as a marker of atherosclerosis and is a good predictor of future vascular events (Lorenz et al., 2007). To ensure the accuracy of IMT measurements, it is necessary to meet the technical, methodological, and operator related criteria (Gonzalez et al., 2008; Stein et al., 2009; Dogan et al., 2010; Society of Atherosclerosis Imaging and Prevention Developed in collaboration with the International Atherosclerosis Society, 2011; Touboul et al., 2012). Thanks to these criteria, the validity and reproducibility of IMT measurement are sufficient and IMT measurement is widely used in clinical practice as well as in the research, and is implemented in several guidelines for cardiovascular risk assessment (National Cholesterol Education Program (NCEP) Expert Panel on Detection, Evaluation, and Treatment of High Blood Cholesterol in Adults (Adult Treatment Panel III), 2002; de la Sierra et al., 2009; Stein et al., 2009; Greenland et al., 2010; Society of Atherosclerosis Imaging and Prevention Developed in collaboration with the International Atherosclerosis Society, 2011).

Atherosclerotic plaque is defined as a focal structure at a vessel wall protruding into the arterial lumen showing a thickness of more than 1.5 mm from the adventitia–media interface (Touboul et al., 2012). Number, proportions, and location of plaques as well as the presence of carotid stenosis caused by plaques need to be considered in the assessment of carotid atherosclerosis severity. The severity of carotid stenosis is quantified according to the flow velocities in the stenosis, residual lumen, and internal carotid artery/common carotid artery flow velocities ratio (Grant et al., 2003). There was a great emphasis on the standardization of the stenosis assessment for the reason of legitimate indication of carotid endarterectomy. The validity and reproducibility of the examination are sufficient for clinical as well as research purposes as long as the technical and personnel conditions are fulfilled (Mohler et al., 2012).

Epidemiological studies have evidenced that AD and VD share common risk factors, which include vascular risk factors such as hypertension, smoking, diabetes mellitus, and hypercholesterolemia (Casserly and Topol, 2004; Gorelick, 2004). These risk factors are also the principal risk factors of atherosclerosis (Greco et al., 2013); considering this, we would expect higher prevalence of large vessel disease in AD than in general population. The results of two large substudies of the prospective cohort of population-based Rotterdam study are in accordance with this hypothesis (Hofman et al., 1997; van Oijen et al., 2007). Both of these substudies were focused on IMT and the degree of carotid (and generally peripheral) atherosclerosis in demented, both AD and vascular dementia (VD), and non-demented subjects. The cross-sectional analysis indicated that the more prominent carotid atherosclerosis the higher probability of dementia. This finding applies for VD, where the association is strong because the atherosclerosis is the principle of the dementia itself, as well as for AD. The longitudinal analysis included measurements at baseline and after 7–9 years and showed that the increased IMT is in the short-term period associated with an increased risk of developing AD. Due to the increased mortality in population with increased IMT, the effect was attenuated in the long-term follow-up (van Oijen et al., 2007). No difference in carotid atherosclerosis is found between AD and high vascular risk patients with VD. This implies that a certain level of impairment is present in both. Concerning the IMT, considered the incipient form of atherosclerosis, also no difference between AD and VD patients was found (Morovic et al., 2009).

In this context, the studies of cognitive decline in asymptomatic carotid stenosis are very interesting. High-degree carotid stenosis (70–99%) or carotid occlusion can be associated with cognitive decline in patients without otherwise clinically evident cerebrovascular disease, making the term “asymptomatic” stenosis somewhat arguable (Johnston et al., 2004; Balucani et al., 2012; Chang et al., 2013). The severity of impairment depends on the quality of collateral blood supply; the character of cognitive decline is influenced by the side of stenosis given by the distinctive functions of cerebral hemispheres. In left-sided stenosis, the verbal memory impairment is more frequent, and in right-sided stenosis there is more prominent visuospatial deficit (Balucani et al., 2012; Zavoreo et al., 2013). Two possible processes are considered in the pathophysiology – silent microembolism or hypoperfusion (Sztriha et al., 2009; Demarin et al., 2012). The carotid endarterectomy or carotid stenting and following reperfusion can improve the mental functions; on the other hand, during both procedures, the microembolism and hypoperfusion can occur as well and cause worsening of the cognitive decline.

Although the IMT in AD is generally increased as compared to healthy population, it does not correlate with cognitive performance in cross-sectional trials (Modrego et al., 2008). In longitudinal studies, IMT and atherosclerosis severity in AD patients correlate with the progression of cognitive impairment (Silvestrini et al., 2009). The progression is faster in AD patients with higher degree of carotid stenosis than in AD patients without stenosis (Silvestrini et al., 2011). Abnormal values of IMT also significantly increase the risk of conversion from amnestic MCI to AD (Viticchi et al., 2012). In a longitudinal study involving a 6-month galantamine treatment of AD, the patients with lower values of IMT at baseline had better response to treatment (Modrego et al., 2009), which suggests that AD patients with lower cerebrovascular burden have slower progression of disease. Details of ultrasound projects focused on IMT and carotid atherosclerosis in AD are listed in Table 1.

**Table 1:** **IMT and carotid atherosclerosis**.

Reference	Aim of study	Type of study	n MCI	n AD	n VD	n Controls	Parameters	Outcome
Hofman et al. (1997)	Frequency of dementia and its subtypes in relation to atherosclerosis and apo-E	Cross-sectional		207	50	1698	IMT and atherosclerotic plaques in CCA and ICA, ankle and brachial systolic pressure	The risk of dementia of any type increases with the severity of atherosclerosis
Modrego et al. (2008)	Correlation of cognitive decline, WML and IMT in AD	Cross-sectional		51			Neuropsychological tests, WML on MRI, IMT in CCA	No correlation of clinical scales with WML or IMT
Modrego et al. (2009)	Association of IMT and response to ACHEI treatment in AD	Longitudinal		50			IMT in CCA and neuropsychological tests at time 0 and after 6 months while on galantamine treatment	Better response to galantamine treatment in lower IMT
Morovic et al. (2009)	Difference in IMT, beta stiffness index and CCA diameter between AD and VD	Cross-sectional		16	22		IMT, beta stiffness index and lumen diameter in CCA	No significant difference in any parameter between AD and VD
Purandare et al. (2005)	Frequency of cerebral emboli, v-a circulation shunts and carotid artery disease in dementia and controls	Cross-sectional		24	17	16	Spontaneus cerebral emboli in MCAs, bubbles in MCAs, PSV in ICA	More cerebral microemboli in VD than controls, in AD not significant, no difference in v-a shunt or carotid stenosis between dementia and controls
Silvestrini et al. (2009)	Correlation of carotid atherosclerosis progression and cognitive impairment in AD	Longitudinal		66			Carotid plaques, flow velocities, PI and IMT in CCA in time 0 and 12 month, while treated with galantamine	Significant correlation of cognitive decline with baseline IMT, IMT change, PI change, antihypertensive drugs
Silvestrini et al. (2011)	Association of ICA stenosis with cognitive decline progression in AD	Longitudinal		411			ICA plaques and flow velocities at baseline and in 12 months	Faster progression of cognitive decline in severe stenosis
van Oijen et al. (2007)	Association of atherosclerosis with dementia subtypes	Longitudinal		476	78		IMT and plaques in CCA and ICA	Higher IMT associated with greater risk of AD
Viticchi et al. (2012)	Association of carotid atherosclerosis and cerebrovascular reactivity with the risk of conversion from MCI to AD	Longitudinal	117	21			IMT and plaques in CCA, BHI in MCAs	Association of higher IMT and lower BHI with faster progression from MCI to dementia

*ACHEI, acetylcholine esterase inhibitor; AD, Alzheimer’s disease; BHI, breath holding index; CCA, common carotid artery; ICA, internal carotid artery; IMT, intima-media thickness; MCA, middle cerebral artery; MCI, mild cognitive impairment; MRI, magnetic resonance imaging; PI, pulsatility index; PSV, peak systolic velocity; v-a, venous-to-arterial; VD, vascular dementia; WML, white matter lesions*.

### Total CBF

Total CBF can be assessed by ultrasonography when measuring flow velocities in carotid and vertebral arteries and multiplying the result by the cross-sectional area of the vessels (average of systolic and diastolic areas). The results gained by this method are comparable to nitrous oxide and SPECT measurements giving the average CBF in a healthy subject of approximately 54 ml/100 g/min (Schoning et al., 1994). Insignificant errors in the measurement of flow velocities and vessel diameter can give significant errors in the final blood flow, up to 10%, but the accuracy and reproducibility of measurement are acceptable when repeated measurements are done and the average is calculated (Gill, 1985; Schoning and Scheel, 1996).

Total CBF reduces with age (Dorfler et al., 2000) and brain parenchymal volume (van Es et al., 2010). According to ultrasound studies, the total CBF is significantly lower in both AD and VD than in healthy controls of the same age (Maalikjy Akkawi et al., 2003; Schreiber et al., 2005; Doepp et al., 2006). This corresponds with changes described for an ICA flow curve in AD, where both systolic and diastolic velocities are lower compared to healthy individuals (Gusti et al., 2004). The association of total CBF with percentage of brain atrophy is weak (van Es et al., 2010). In an ultrasound study of three groups of patients with documented cerebral atrophy (AD, VD, and cognitively normal subjects), the total CBF was significantly lower in patients with dementia than in those without a cognitive impairment. There was no significant difference between two types of dementia (Albayrak et al., 2006). Details of ultrasound projects focused on total CBF in AD are listed in Table 2.

**Table 2:** **Total cerebral blood flow**.

Reference	Aim of study	Type of study	n MCI	n AD	n VD	n Controls	Parameters	Outcome
Albayrak et al. (2006)	Comparison of cerebral blood flow in demented (AD, VD) and cognitively normal subjects, both with brain atrophy	Cross-sectional		9	9	10	Flow velocities and cross-sectional area of the vessel in ICAs and VAs	Total, anterior and right CBF lower in dementia, no difference between two types of dementia
Doepp et al. (2006)	Possible differentiation of AD and VD by various extra- and intracranial ultrasound parameters	Cross-sectional		20	20	12	Flow velocities and PI in MCAs, flow velocities and cross-sectional area in ICAs and VAs, cerebral circulation time, global cerebral blood volume	No significant difference in trans- and extracranial ultrasound between AD and VD
Gusti et al. (2004)	Comparison of carotid flow velocities and flow curve in AD and controls	Cross-sectional		18		40	Flow velocities in carotid arteries	Lower cerebral vascular filling in AD
Maalikjy Akkawi et al. (2003)	Possibility of CBF volume assessment by TCD, difference between AD and controls, correlation with cognitive decline	Cross-sectional		50		50	Flow velocities and vessel diameter in ICA and VA, calculation of cerebral blood flow	Decrease in CBF volume in AD compared to controls, positive correlation between dementia severity and CBF
Schreiber et al. (2005)	CBF, cerebral circulation time and cerebral blood volume in AD, VD and controls	Cross-sectional		20	20	12	Flow velocity and cross-sectional area of ICA and VA, time of contrast agent transfer from ICA to IJV	Difference in CBF and transit time between dementia and controls, no difference in CBF volume or between AD and VD

*AD, Alzheimer’s disease; CBF, cerebral blood flow; ICA, internal carotid artery; IJV, internal jugular vein; MCA, middle cerebral artery; MCI, mild cognitive impairment; PI, pulsatility index; TCD, transcranial Doppler; VA, vertebral artery; VD, vascular dementia*.

## Transcranial Ultrasound in AD

### Flow velocities, cerebrovascular resistance, and cerebrovascular reserve capacity

The CBF curve in a transcranial ultrasound examination is characterized by two main flow velocities – peak systolic velocity and end diastolic velocity. These velocities can be measured in all major intracranial vessels – anterior, middle, and posterior cerebral arteries, vertebral arteries; and basilar artery. The mean flow velocity and indices describing the resistance of intracranial vessels can be derived from the flow curve. The reproducibility of flow velocities measurement is good when done by an experienced examiner (McMahon et al., 2007).

Many studies have found significantly lower flow velocities in AD compared to controls (Caamano et al., 1993; Roher et al., 2006, 2011; Sun et al., 2007; Vicenzini et al., 2007; Claassen et al., 2009; Stefani et al., 2009; Gucuyener et al., 2010). The most often studied vessel was the middle cerebral artery (MCA) while other major intracranial arteries were studied less frequently. The most often decreased velocity in MCA in AD patients compared to healthy controls was the mean flow velocity (Roher et al., 2006, 2011; Vicenzini et al., 2007; Claassen et al., 2009; Stefani et al., 2009), although not all results support these findings (Ries et al., 1993). Decreases in peak systolic and end diastolic velocities varied in different arteries (Caamano et al., 1993; Sun et al., 2007; Gucuyener et al., 2010). According to a large longitudinal study (Ruitenberg et al., 2005), subjects with higher velocities in MCA were less likely to develop AD. The question is – why the decrease should be most prominent in the MCA. We speculate this could be a consequence of pathological changes in AD, where temporal and parietal lobes supplied by MCA are most affected. In this context, a comparison of healthy subjects and patients with MCI would be interesting, but the results are ambiguous (Roher et al., 2011). No significant difference in flow velocities was found between AD and VD, neither there is a significant side asymmetry.

Unlike SPECT, the transcranial Doppler measures only flow velocities and not absolute blood flow, and the assessment of flow velocities is not helpful in an individual patient due to the wide range of normal values of flow velocities. The methods for assessment of regional cerebral perfusion or metabolism (SPECT, PET) have high sensitivity and specificity in distinguishing AD vs. normal controls (depending on the stage of the disease and method employed) based on characteristic perfusion or metabolism reduction in temporoparietal association cortex: SPECT can reach sensitivity of 65–96% and specificity of 80–87%, PET can reach even sensitivity of 93–94% and specificity of 63–73% (Wollman and Prohovnik, 2003; Matsuda, 2007). These two methods can be used to make the clinical diagnosis of AD more accurate in some unclear cases.

A parameter describing autoregulation of cerebral perfusion is cerebrovascular reserve capacity, which reflects the capability of brain microvasculature to regulate cerebral perfusion in a reaction to various stimuli, thanks to constriction or dilatation. The most often used stimulus is a change of the arterial CO_2_ level that can be induced using breath holding, CO_2_ inhalation, or intravenous acetazolamide injection. Other less often used stimuli include hand movement, cognitive exercise, or blood pressure challenge (i.e., physical exercise). The cerebrovascular reserve capacity is expressed as the ratio of mean flow velocity in basal conditions and mean flow velocity in the conditions of a higher CO_2_ level. In normal brain, there is an increase in flow velocities. When breath holding is the stimulus, the ratio can be multiplied by the duration of breath holding and expressed as breath holding index (BHI). Cerebrovascular reserve capacity decreases with age (Peisker et al., 2010).

The cerebrovascular reserve capacity is in clinical practice routinely tested before revascularization procedures in carotid stenosis or occlusion. The established methods for cerebrovascular reserve capacity assessment are scintigraphic techniques such as SPECT and PET with the use of various radioactive tracer compounds, all of them evaluating the cerebral perfusion in basal conditions and after vasodilatory stimulus (acetazolamide injection or CO_2_ inhalation). In comparison with these direct techniques, the transcranial Doppler examination is an indirect assessment based on the relative increase in flow velocities after vasodilatory stimulus (usually acetazolamide injection, CO_2_ inhalation or breath holding). All three transcranial Doppler methods correlate very well to 133Xe SPECT (Bishop et al., 1986; Dahl et al., 1992; Muller et al., 1995) with the breath-holding method being the less accurate but sufficient for first screening examination (Markus and Harrison, 1992; Muller et al., 1995). Compared to scintigraphic techniques the ultrasound examination is non-invasive and inexpensive.

Concerning the cerebrovascular reserve capacity measured by transcranial Doppler in the MCA in AD patients, the results are more consistent than those for solely flow velocities. In AD patients, the reactivity to different stimuli in the MCA is significantly lower than in healthy controls (Provinciali et al., 1990; Bar et al., 2007; Lee et al., 2007; Vicenzini et al., 2007; Stefani et al., 2009). Only some studies with fewer subjects do not fully support these findings (Matteis et al., 1998; Claassen et al., 2009). In one of these studies, the result could be influenced by the selection of very mild AD cases (MMSE 25) (Claassen et al., 2009). In another study, it is not sufficiently described how the cognitive impairment was ruled out in control subjects (Matteis et al., 1998). One study proved a better cerebrovascular reserve capacity in AD than VD, but the result was not statistically significant (Likitjaroen et al., 2009). Healthy subjects with higher cerebrovascular reserve capacity are less likely to develop a cognitive decline (AD or VD) (Ruitenberg et al., 2005). Although the impairment of cerebrovascular reserve capacity is more serious in VD, it seems that the microvasculature is altered in both main types of dementia (Bar et al., 2007; Vicenzini et al., 2007).

On the other hand, the hypercapnia challenge in SPECT and PET studies give ambiguous results without convincing evidence of decreased cerebrovascular reserve capacity in AD (Yamaguchi et al., 1980; Bonte et al., 1989; Kuwabara et al., 1992; Stoppe et al., 1995; Knapp et al., 1996; Jagust et al., 1997; Oishi et al., 1999; Pavics et al., 1999). However, it must be taken into account that in earlier publications, the diagnostic criteria for AD may differ from nowadays criteria and older devices may not give very accurate results (Glodzik et al., 2013).

Again the comparison with asymptomatic carotid stenosis or occlusion is interesting. In cases of high degree stenosis or occlusion with insufficient collateral blood supply, the chronic hypoperfusion exhausts the cerebrovascular reserve. This can be observed in different examination methods (Oka et al., 2013) including transcranial Doppler ultrasound examination using BHI (Balestrini et al., 2013; Zavoreo et al., 2013). The decrease of cerebrovascular reserve capacity correlates with the cognitive decline (Zavoreo et al., 2013).

The cerebrovascular reserve capacity of posterior cerebral artery in reaction to a visual stimulus was often tested. The function of occipital lobe should be preserved until late stages of AD. The results of such projects were ambiguous (Asil and Uzuner, 2005; Rosengarten et al., 2006, 2007; Gucuyener et al., 2010) and, thus, not differentiating AD from VD.

The reason for the decreased cerebrovascular reserve capacity is not entirely clear. In VD, the cause is probably a small vessel disease. In AD, amyloid deposits represent the likely culprit – in cerebral amyloid angiopathy, the cerebrovascular reserve capacity is also compromised (Menendez-Gonzalez et al., 2011). Another hypothesis suggests the role of insufficient acetylcholine production necessary for vasodilatation. Therapeutic tests with acetylcholine inhibitors (galantamine or donepezil) demonstrated an increase in flow velocities and improvement of vessel reactivity in both VD and AD (Rosengarten et al., 2006; Bar et al., 2007; Ghorbani et al., 2010). In longitudinal follow-up studies, the BHI significantly correlated with neuropsychological tests – MMSE and Alzheimer’s Disease Assessment Scale-cognitive subscale (ADAS-Cog) in AD (Silvestrini et al., 2006). MCI patients with pathological values of BHI have greater risk of converting to dementia than patients with normal values (Viticchi et al., 2012). Details of ultrasound projects focused on flow velocities and cerebrovascular reserve capacity in AD are listed in Table 3.

**Table 3:** **Flow velocities, cerebrovascular resistance, and cerebrovascular reserve capacity**.

Reference	Aim of study	Type of study	n MCI	n AD	n VD	N Controls	Parameters	Outcome
Asil and Uzuner (2005)	Assessment of CVRC in the occipital lobe in AD	Cross-sectional		15	12	9	Flow velocities in PCAs during eyes opened and eyes closed	No significant difference neither in flow velocities at rest nor at stimuli in three groups; decreased reactivity in VD at stimulus
Bar et al. (2007)	CVRC in AD compared to VD and healthy controls, reactivity after ACHEI treatment	Cross-sectional Longitudinal		17	17	20	Flow velocities in MCA at rest and after CO_2_ inhalation in AD and VD repeated after 5 weeks of galantamine treatment	CVRC in MCA decreased in AD and VD in comparison to healthy controls, better CVRC after galantamine treatment on both AD and VD
			
Caamano et al. (1993)	Comparison of flow velocities in MCA and BA in AD, VD and controls	Cross-sectional		12	12	12	Flow velocities in right and left MCA and BA	Decreased values in demented patients
Claassen et al. (2009)	Assessment of cerebral hemodynamics impairment in early stage AD	Cross-sectional		9		8	Flow velocities in MCA, blood pressure, cerebrovascular resistance index	Significantly reduced flow velocities and increased resistance in AD
Ghorbani et al. (2010)	Assessment of the effect of Donepezil on cerebral blood flow velocity in AD patients	Longitudinal		11			Flow velocities in PCA and MCA at baseline, after 4 weeks of donepezil 5 mg and after another 4 weeks of donepezil 10 mg	Increase in PSV and MFV in MCA, and MFV and EDV in PCA after 10 mg treatment
Gucuyener et al. (2010)	CVRC in PCAs in AD compared to depressive pseudo-dementia	Cross-sectional		11	13	10	Flow velocities in both PCAs simultaneously; in steady state and after a visual stimulus	Lower flow velocities at rest and after stimulus in both AD and depressive pseudodementia then controls. CVRC impaired in AD, not in depressive pseudodementia
Lee et al. (2007)	Assessment of CVRC in AD	Cross-sectional		17		17	Flow velocities and PI in MCA bilaterally in normal conditions and after 5 min of rebreathing	No difference in baseline MFV and PI between subjects and controls, CVRC significantly decreased on both sides in AD
Likitjaroen et al. (2009)	Comparison of CVRC in AD and VD	Cross-sectional		9	9		Flow velocities in MCA in normal conditions and after 1000 mg acetazolamide i.v.	Non-significantly better CVRC in AD than VD
Matteis et al. (1998)	Comparison of CVRC in AD and VD	Cross-sectional		10	10	20	Flow velocities in MCA during apnea, hand movement and verbal and design discrimination	CVRC to apnea lower in VD; hand movement – contralateral increase in flow in AD and controls, bilateral in VD; bilateral response on cognitive stimuli in AD and VD, corresponding side response in controls
Provinciali et al. (1990)	Comparison of CVRC in AD, VD and controls	Cross-sectional		20	20	25	Flow velocities in MCA at rest, after hyperventilation, apnea and 5 min air rebreathing	Higher PI, lower velocity decrease in hyperventilation in both dementias; rest flow velocities and response to hypercapnia lower in VD than AD or controls
Ries et al. (1993)	Utility of TCD in differentiation of AD and multi-infarct dementia	Cross-sectional		24	17	64	PSV and EDV in all large intracranial vessels bilaterally, pulse curve in MCA	No difference in PSV in all three groups, difference in MFV, EDV and effective pulsatility range in VD compared to AD or controls
Roher et al. (2006)	Comparison of mean flow velocities and PI in intracranial arteries in AD and controls	Cross-sectional		25		30	Flow velocities in 16 different segments of circle of Willis	Higher PIs in AD, non-significantly lower mean flow velocities in AD
Roher et al. (2011)	Utility of TCD in diagnosing and preventing AD	Cross-sectional	11	42		50	Flow velocities in 16 different segments of circle of Willis	Significant difference in MFV and PI in left siphon, left ICA and right distal MCA between AD and controls
Rosengarten et al. (2006)	Influence of ACHEI treatment on vasoregulation in AD	Longitudinal		8		16	Flow velocities in PCA and MCA in rest and at stimulation (text reading) at baseline, after 4 weeks of donepezil 5 mg and after another 4 weeks of donepezil 10 mg	Decrease in attenuation parameter after 10 mg in AD = dose dependent resolution of functional vascular deficit
Rosengarten et al. (2007)	Comparison of activation-flow coupling in AD, VD and controls	Cross-sectional		15	10	15	Flow velocities in PCA and MCA in rest and at stimulation (text reading)	Lower increase in PSV in VD
Ruitenberg et al. (2005)	Correlation of flow velocities with cognitive decline and hippocampal atrophy	Cross-sectional		13	1	1718	Flow velocities in MCAs at rest and after 5 min of 5% CO_2_	Greater PSV, MFV, EDV – less likely dementia and bigger hippocampus and amygdala No association of CVRC and presence of dementia
Silvestrini et al. (2006)	Influence of cerebral hemodynamics alterations on the evolution of cognitive impairment	Longitudinal		53			Flow velocities in MCAs at rest and after breath-holding, time 0 and 12 month, during this time donepezil 5 mg daily for 3 month, then 10 mg daily	Positive correlation of neuropsychological tests changes with BHI, age and DM
Silvestrini et al. (2009), Stefani et al. (2009)	Comparison of cerebral hemodynamics in AD and controls	Cross-sectional		40		40	Flow velocities, PI and BHI in MCA	Lower MFV, higher PI and lower BHI in MCA in AD than in controls
Sun et al. (2007)	Changes in cerebral flow velocities in MCI and controls	Cross-sectional	30			30	Flow velocities in MCA, ACA, BA	Decreased PSV, MFV and EDV in MCA and ACA in MCI compared to controls
Vicenzini et al. (2007)	Comparison of flow velocities, PI and CVRC in AD, VD, and controls	Cross-sectional		60	58	62	Flow velocities in MCA in normal conditions, after hyperventilation and CO_2_ inhalation	Lower MFV, higher PI and lower CVRC in AD and VD compared to controls
Viticchi et al. (2012)	Association of carotid atherosclerosis and cerebrovascular reserve capacity with the risk of conversion from MCI to AD	Longitudinal	117	21			IMT and plaques in CCA, BHI in MCAs	Association of higher IMT and lower BHI with faster progression from MCI to dementia

*ACA, anterior cerebral artery; ACHEI, acetylcholine esterase inhibitor; AD, Alzheimer’s disease; BA, basilar artery; CAA, cerebral amyloid angiopathy; CVRC, cerebrovascular reserve capacity; DM, diabetes mellitus; EDV, end diastolic velocity; ICA, internal carotid artery; MCA, middle cerebral artery; MCI, mild cognitive impairment; MFV, mean flow velocity; PCA, posterior cerebral artery; PI, pulsatility index; PSV, peak systolic velocity; VD, vascular dementia*.

### Spontaneous cerebral microembolization and paradoxical embolization via right–left shunts

Recent evidence suggests that cerebral microemboli can lead to a cognitive decline (Pugsley et al., 1994; Gaudet et al., 2009). Cerebral microemboli can originate from arterial sources or venous sources in setting of right–left shunts (intracardiac – foramen ovale patens, atrial septal defects). The spontaneous cerebral embolization can be monitored using a headframe with attached ultrasound probes for time periods of usually 1–24 h. Right–left shunts are examined by intravenous injection of a microbubble agent (agitated saline or hydroxyethyl starch) and observing the presence of microbubbles in brain vessels using transcranial ultrasound at rest and during the Valsalva maneuver. The accuracy of right–left shunt assessment by transcranial Doppler ultrasound compared to the transesophageal echocardiography as a gold standard ranges from 68 to 100% according to the reports in literature, some of them claiming the transcranial Doppler method even more accurate (Nemec et al., 1991; Teague and Sharma, 1991; Di Tullio et al., 1993; Jauss et al., 1994; Job et al., 1994; Sastry et al., 2009). The sensitivity and reproducibility of the examination is highest when performed repeatedly (twice) with the use of Valsalva maneuver (Droste et al., 1999).

There were not many studies focused on spontaneous cerebral embolization in AD. One work suggested that it is more frequent in patients with AD or VD than in healthy controls (Purandare et al., 2005). This suggestion was later confirmed by a larger case–control study (Purandare et al., 2006). In this particular project, there was no significant difference in the incidence of carotid artery atherosclerosis – i.e., possible source of microembolization. Prevalence of patent foramen ovale in AD and VD cohort was 33% in this study, which is higher than usually reported 20–25% in general population (Hara et al., 2005), but no larger epidemiological studies of prevalence in AD were done. The same author found the association of spontaneous cerebral microembolization with more rapid cognitive decline in dementia (Purandare and Burns, 2009). Details of ultrasound projects focused on spontaneous and paradoxical embolization in AD are listed in Table 4.

**Table 4:** **Spontaneous cerebral microembolization and paradoxical embolization via right–left shunts**.

Reference	Aim of study	Type of study	n MCI	n AD	n VD	n Controls	Parameters	Outcome
Purandare et al. (2005)	Spontaneous cerebral microemboli, v-a circulation shunts and carotid artery disease in dementia and controls	Cross-sectional		24	17	16	Spontaneus cerebral emboli in MCAs, bubbles in MCAs, PSV in ICA	More cerebral microemboli in VD than controls, in AD not significant, no difference in shunt or carotid stenosis between dementia and controls
Purandare et al. (2006)	Spontaneous cerebral microemboli, v-a circulation shunts and carotid artery disease in dementia and controls	Cross-sectional		85	85	150	Spontaneus cerebral emboli in MCAs, bubbles in MCAs, PSV in ICA	More cerebral microemboli in VD and AD than controls, no difference in shunt or carotid stenosis between dementia and controls
Purandare and Burns (2009)	Association of spontaneous cerebral microembolization with dementia etiology, dementia progression and depression in dementia or controls	Cross-sectional Longitudinal		85	85	150	Spontaneus cerebral emboli in MCAs, bubbles in MCAs, PSV in ICA. Neuropsychological tests in time 0 and 6 months	More cerebral microemboli in AD and VD than controls, more in depression (both dementia and controls). Association with more rapid cognitive decline in dementia

*AD, Alzheimer’s disease; ICA, internal carotid artery; MCA, middle cerebral artery; MCI, mild cognitive impairment; PSV, peak systolic velocity; v-a, venous-to-arterial; VD, vascular dementia*.

## Conclusion

The current evidence suggests that the brain perfusion in AD patients, in general, is impaired compared to healthy non-demented population. The most prominent ultrasonographical findings in extracranial circulation in AD patients show an increased IMT and higher burden of carotid artery atherosclerosis. The most often identified changes in intracranial circulation are lower flow velocities, lower total CBF (not explained by brain atrophy only), and most notably impaired cerebrovascular reserve capacity (Table 5). These findings seem to be valid for both AD and VD.

**Table 5:** **Neurosonological parameters in AD – summary**.

Ultrasound parameter	Findings in AD	Conclusion
IMT	Increased IMT associated with increased short-term risk of developing AD, converting from MCI to AD, and lower response to galantamine treatment of AD	In combination with other neurosonological methods and vascular risks assessment can help to identify patients in higher risk of faster progression of AD
	Correlates with the progression of AD	
Carotid atherosclerosis	Higher degree of carotid atherosclerosis associated with increased short-term risk of developing AD and converting from MCI to AD	In combination with other neurosonological methods and vascular risks assessment can help to identify patients in higher risk of faster progression of AD
	Correlates with the progression of AD	
Total cerebral blood flow	Decreased in AD	Inconclusive
	Not dependent on brain atrophy	
	Longitudinal data not available	
Flow velocities	Variably decreased MFV in MCA in AD	Inconclusive
	Decreased flow velocities associated with increased risk of developing AD	
Cerebrovascular reserve capacity	Decreased in AD	Best correlation with AD incidence and progression among all neurosonological parameters
	Decreased CVRC associated with increased risk of developing AD	

*AD, Alzheimer’s disease; IMT, intima-media thickness; MCA, middle cerebral artery; MCI, mild cognitive impairment; MFV, mean flow velocity*.

Ultrasonography of extra- and intracranial brain vessels can be helpful in AD patients to identify individuals who are in a higher risk of disease progression. Ultrasonography can be also useful for stratification of MCI patients and can contribute to predict the risk of conversion to AD. The vascular risk factors surveillance and treatment in preclinical stages of AD is of great clinical importance and it could help to delay the development of cognitive decline in susceptible individuals. Ultrasonography is not especially beneficial in differentiating AD and VD, because the microvasculature is altered in both types of dementia.

## Author Contributions

Barbora Urbanova, Ales Tomek, Robert Mikulik, and Jakub Hort took part in designing the aim and scope of the review. Barbora Urbanova, Ales Tomek, and Hana Magerova did the literature search. All the authors took part in detailed study and interpretation of the reviewed articles. All the authors took part in writing various sections of this article. Barbora Urbanova, Jakub Hort, Robert Mikulik, and Ales Tomek reviewed whole article.

## Conflict of Interest Statement

The authors declare that the research was conducted in the absence of any commercial or financial relationships that could be construed as a potential conflict of interest.
